# Supplementary material for: Serum procalcitonin level is independently associated with mechanical ventilation and case-fatality in hospitalized COVID-19-positive US veterans–A potential marker for disease severity
Source: PLoS One. 2023 Apr 17;18(4):e0284520. doi: 10.1371/journal.pone.0284520 (PMC10109491; doi:10.1371/journal.pone.0284520)
Supplement: S1 Table — (DOCX) [file pone.0284520.s001.docx]

|  | **Total** | **Procalcitonin**  **≤ 0.2** | **Procalcitonin**  **>0.2** | **p-value** |
| --- | --- | --- | --- | --- |
| Number of Covid-19 positive veterans (n) | 27,154 | 16,135 | 11,019 |  |
| PCT (ng/ml) (IQR) | 0.2 (0.1-0.4) | 0.1 (0.1-0.1) | 0.5 (0.3-1.1) | <0.001 |
| CRP (mg/dL) | 14.1 (5.6-55.3) | 10.7 (4.1-40.0) | 19.7 (9.4-91.4) | <0.001 |
| Quartiles of CRP (mg/dL) |  |  |  | <0.001 |
| < 1.8 | 1,486 ( 5.5%) | 1,221 ( 7.6%) | 265 ( 2.4%) |  |
| 1.8 to < 7.3 | 3,653 (13.5%) | 2,673 (16.6%) | 980 ( 8.9%) |  |
| 7.3 to < 25.1 | 5,419 (20.0%) | 2,920 (18.1%) | 2,499 (22.7%) |  |
| >= 25.1 | 5,880 (21.7%) | 3,040 (18.8%) | 2,840 (25.8%) |  |
| Unknown/missing | 10,716 (39.5%) | 6,281 (38.9%) | 4,435 (40.2%) |  |
| WBC (K/cmm) | 6.5 (4.9-8.9) | 6.1 (4.7-8.1) | 7.3 (5.3-10.2) | <0.001 |
| Quartiles of WBC (K/cmm) |  |  |  | <0.001 |
| < 5.9 | 10,108 (37.2%) | 6,817 (42.2%) | 3,291 (29.9%) |  |
| 5.9 to < 7.6 | 5,386 (19.8%) | 3,406 (21.1%) | 1,980 (18.0%) |  |
| 7.6 to < 9.9 | 4,392 (16.2%) | 2,449 (15.2%) | 1,943 (17.6%) |  |
| >= 9.9 | 4,691 (17.3%) | 1,991 (12.3%) | 2,700 (24.5%) |  |
| Unknown/missing | 2,577 ( 9.5%) | 1,472 ( 9.1%) | 1,105 (10.0%) |  |
| Ddimmer (mg/L FEU) | 0.9 (0.5-1.7) | 0.8 (0.4-1.4) | 1.1 (0.6-2.2) | <0.001 |
| Quartiles of Ddimmer (mg/L FEU) |  |  |  | <0.001 |
| < 0.4 | 3,509 (18.5%) | 2,495 (22.3%) | 1,014 (13.1%) |  |
| 0.4 to < 0.7 | 4,394 (23.2%) | 2,920 (26.1%) | 1,474 (19.0%) |  |
| 0.7 to < 1.5 | 5,590 (29.5%) | 3,304 (29.5%) | 2,286 (29.5%) |  |
| >= 1.5 | 5,448 (28.8%) | 2,464 (22.0%) | 2,984 (38.5%) |  |
| Ferritin (ng/mL) | 500.2 (235.0-1008.0) | 408.5 (193.6-795.0) | 696.2 (335.0-1355.0) | <0.001 |
| Quartiles of Ferritin (ng/mL) |  |  |  | <0.001 |
| < 89.5 | 1,406 ( 7.5%) | 1,085 ( 9.7%) | 321 ( 4.3%) |  |
| 89.5 to <236.7 | 3,293 (17.7%) | 2,358 (21.1%) | 935 (12.5%) |  |
| 236.7 to <567.5 | 5,470 (29.3%) | 3,556 (31.9%) | 1,914 (25.5%) |  |
| >=567.5 | 8,478 (45.5%) | 4,151 (37.2%) | 4,327 (57.7%) |  |
| Lactate (mmol/L) | 1.5 (1.1-2.1) | 1.4 (1.1-1.9) | 1.7 (1.2-2.3) | <0.001 |
| Quartiles of Lactate (mmol/L) |  |  |  | <0.001 |
| < 1.1 | 3,344 (12.3%) | 2,110 (13.1%) | 1,234 (11.2%) |  |
| 1.1 to < 1.5 | 4,507 (16.6%) | 2,805 (17.4%) | 1,702 (15.4%) |  |
| 1.5 to < 2.1 | 4,712 (17.4%) | 2,571 (15.9%) | 2,141 (19.4%) |  |
| >= 2.1 | 4,485 (16.5%) | 1,987 (12.3%) | 2,498 (22.7%) |  |
| Unknown/missing | 10,106 (37.2%) | 6,662 (41.3%) | 3,444 (31.3%) |  |

Supplementary Table 1:
