# Supplementary material for: Serum procalcitonin level is independently associated with mechanical ventilation and case-fatality in hospitalized COVID-19-positive US veterans–A potential marker for disease severity
Source: PLoS One. 2023 Apr 17;18(4):e0284520. doi: 10.1371/journal.pone.0284520 (PMC10109491; doi:10.1371/journal.pone.0284520)
Supplement: S2 Table — This analysis was performed on 10,813 veterans with at least two serum PCT tests, where a binary serum PCT change variable between the first and last serum PCT tests was created, scored as 0 if decreased (positive result), versus 1 if increased or no change (negative result). 1- unadjusted. Model 2 adjusted for demographic and comorbidity conditions (i.e., adjusted for age, race, BMI, history of CKD, Heart failure, hypertension, liver disease, and diabetes), Model 3 adjusted for demography, comorbidity conditions (Model 2) as well as pneumonia (presence or absence)and antibiotic use (used or not used). Model 4 adjusted for all covariates of Model 3 and lab test results (CRP, WBC, and lactate). (DOCX) [file pone.0284520.s002.docx]

| **Model** | **Subgroups** | **Mechanical ventilation (MV)**  HR (95% CI) | **In-hospital death**  HR (95% CI) |
| --- | --- | --- | --- |
| 1 | Decrease PCT values vs. others | 0.83(0.75, 0.92) | 0.45(0.42, 0.49) |
| 2 | Decrease PCT values vs. others | 0.83(0.75, 0.92) | 0.47(0.44, 0.51) |
| 3 | Decrease PCT values vs. others | 0.82(0.75, 0.91) | 0.47(0.43, 0.50) |
| 4 | Decrease PCT values vs. others | 0.75(0.68, 0.83) | 0.42(0.39, 0.45) |

Supplementary Table 2:
