# Supplementary figures and images for: Serum procalcitonin level is independently associated with mechanical ventilation and case-fatality in hospitalized COVID-19-positive US veterans–A potential marker for disease severity
Source: PLoS One. 2023 Apr 17;18(4):e0284520. doi: 10.1371/journal.pone.0284520 (PMC10109491; doi:10.1371/journal.pone.0284520)

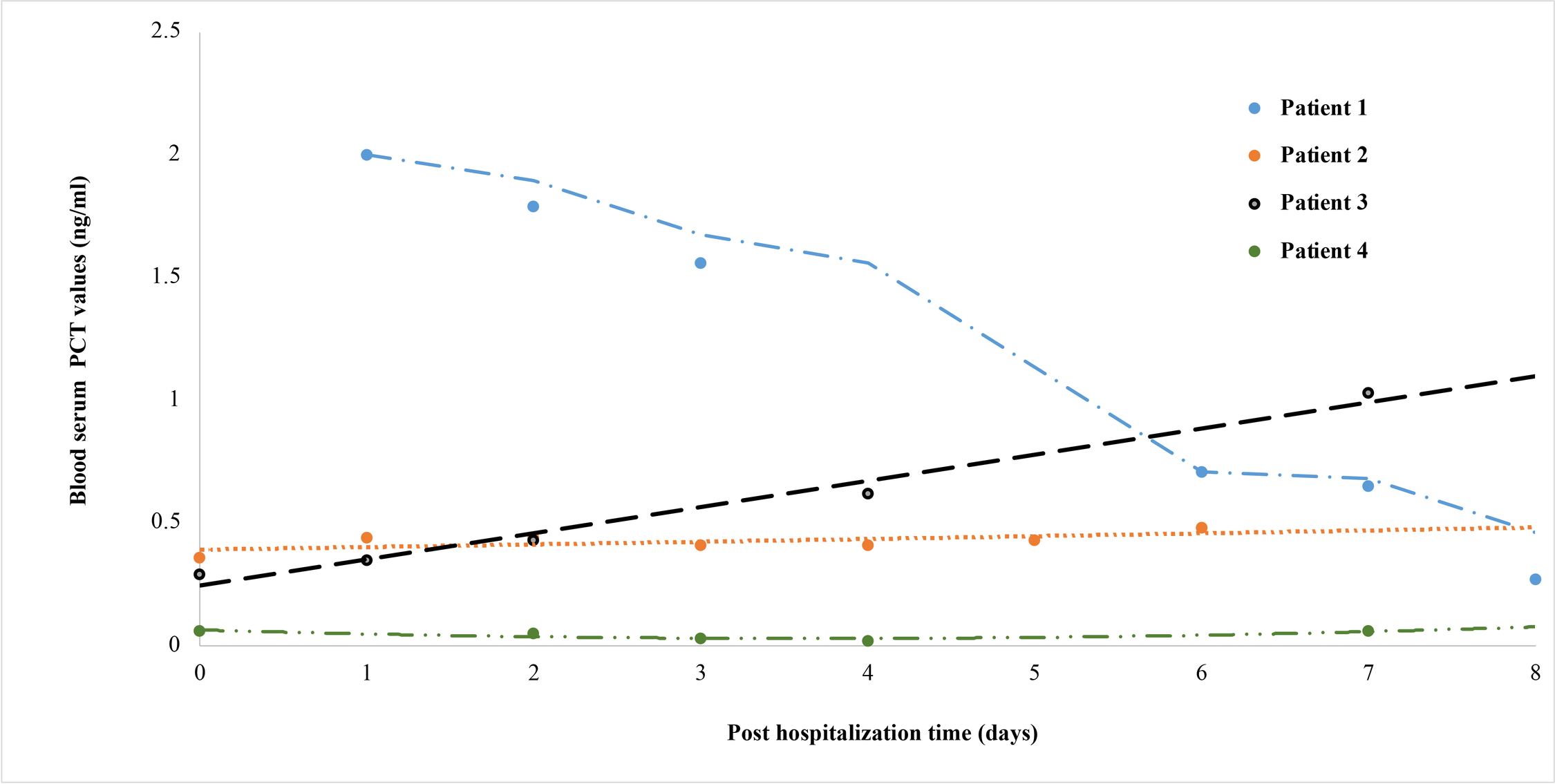

Supplement: S1 Fig — While patients 1 and 4 recovered from the disease, patients 2–3 suffered in-hospital death after receiving the mechanical ventilation treatment. (TIF) [file pone.0284520.s003.tif]
