# Supplementary material for: Serum procalcitonin level is independently associated with mechanical ventilation and case-fatality in hospitalized COVID-19-positive US veterans–A potential marker for disease severity
Source: PLoS One. 2023 Apr 17;18(4):e0284520. doi: 10.1371/journal.pone.0284520 (PMC10109491; doi:10.1371/journal.pone.0284520)
Supplement: Attachment — Submitted filename: Revision Cover Letter.docx [file pone.0284520.s004.docx]

**Editor-In-Chief**

Emily Chenette

Cambridge, England, UK

Dear Dr. Chenette,

Firstly, we would like to express our sincere gratitude for the reviewer’s feedback and the time spent reviewing this manuscript. We have reviewed the comments received and made the appropriate changes. There are given below.

***General***

**Funding statement:** Per the request we changed the funding statement as requested (see below).

“This investigation was supported by an unrestricted investigator-initiated research grant from Gilead Sciences (# CO-US-983-6072). The research was also, in part, supported by the National Center for Advancing Translational Sciences of the National Institutes of Health under Award Number UL1TR002538. The content is solely the responsibility of the authors and does not necessarily represent the official views of the National Institutes of Health or Gilead Scientific Inc. The funders had no role in study design, data collection, and analysis, decision to publish, or preparation of the manuscript”.

**Tables, Figures, References, and Caption**

As requested, the Tables are included within the text, and Figure Captions are inserted where they were mentioned first. The reference style is corrected to the PLOS one format (end note style downloaded from the PLOS one website).

**Reviewer’s comment**

General concern: Reviewer 1 marked “no” to the journal’s question 1 (is the manuscript technically sound, and do the data support the conclusion” that manuscript data?).

Response: We have realized that our hypothesis was not clearly written in the introduction. Thus, we reworded it on Page 4, Lines 110-116. It now reads,

“This study was therefore designed to test the primary hypothesis that there would be an association between elevated serum PCT values and symptom severity in a larger cohort of hospitalized Veterans with COVID-19 who were admitted to the nationwide Veterans Health Administration hospital system. As the secondary goals, we seek to clarify whether or not serial PCT tests would predict deteriorating clinical status in hospitalized veterans with COVID-19 positivity, as well as, whether or not the PCT levels and symptom severity disproportionally affect the Black race over the White race. If the association is replicated in this study, it would further support the pertinent benefit of using PCT to guide the prognosis of worsening COVID-19 disease.”

Reviewer 1 concern 1: **Fig. 1, right panel, the error bar is very high for the last test in the in-hospital mortality group.**

Response: We agree with the reviewer that a very wide error bar, when the error bar represents a standard error or a 95% confidence interval, can be a concern as it suggests the estimate of the statistic being displayed has low precision. It appears that the reviewer glanced at the graph and misinterpreted the lines as error bars. Actually, the graph is a boxplot, where individual observations are being presented, so it is a graph showing the distribution of individual observations, being an alternative to showing a histogram. The line that the reviewer misinterpreted as an error bar is just a way to show what the maximum individual observation is. It is perfectly fine for it to be a large (wide) value, so there is actually no concern at all. We describe what the lines represent in the footnote of the graph, so readers unfamiliar with boxplots interpret them correctly.

Reviewer 1 concern 2: **In this study, in-patients were analyzed, with out-patient, non-COVID patients and healthy control missed, which will make this study based on critically ill cases.**

Response: We agree with the reviewer that observed associations are specific to the patient population studied, and any inference beyond that is an extrapolation, although perhaps reasonable. Ours was a very specific patient population, as the reviewer noted. We added the following sentence to the limitations in the Discussion section (Page 19, lines 445-455)

“It is worth noting that our study sample only included patients hospitalized with COVID-19 whose healthcare providers ordered PCT tests, in which we demonstrated that serial measurements of PCT were associated with subsequent mechanical ventilation and death. Strictly speaking, the observed associations we report are only supported for that specific subgroup, perhaps critically ill, of hospitalized COVID-19 patients whose providers were concerned enough to order PCT tests.”

Also, per the critique, to emphasize that this data was from critically ill patients, we also included hospitalized veterans in the title:

New title: ‘Serum Procalcitonin Level is Independently Associated with Mechanical Ventilation and Case-Fatality in Hospitalized COVID-19-Positive US Veterans – A Potential Marker for Disease Symptom Severity.”

Reviewer 1 concern 3:

PCT has some role in the diagnosis of patients with sepsis. So the specificity of PCT for COVID is a concern. ROC curve analysis is warranted to set up a cutoff and determine corresponding sensitivity and specificity.

Response: We agree with the reviewer. We have made no attempt to determine if PCT is detecting COVID, a comorbid infection, both, or sepsis from either of both of these. A ROC curve analysis is a tool that could help answer this question, either in a diagnostic sense or in the prognostic prediction of the two outcomes we studied. It would be a big effort to do that with the needed thoroughness. We could do a quick ROC analysis, but reporting it could be misleading and so irresponsible. All our study has done is demonstrate that PCT is a candidate variable for further development into a prognostic model. Developing a PCT tool, which would include a prediction score or PCT cutoff, is something that we have already invited others to do in our manuscript. It is common to first publish papers to identify “risk factors” which provide suggestions for others to develop diagnostic or prognostic models using those factors, so our paper is still useful, only taking it as far as we have.

Kind Regards,

Sujee Jeyapalina, PhD

Division of Plastic Surgery, Department of Surgery

University of Utah School of Medicine

Salt Lake City, UT 84132

Email: Sujee.jeyapalina@hsc.utah.edu

Tel: 801-582-1565 ext. 1854

Fax: 801-588-5939
