# Supplementary material for: Serum procalcitonin level is independently associated with mechanical ventilation and case-fatality in hospitalized COVID-19-positive US veterans–A potential marker for disease severity
Source: PLoS One. 2023 Apr 17;18(4):e0284520. doi: 10.1371/journal.pone.0284520 (PMC10109491; doi:10.1371/journal.pone.0284520)
Supplement: Attachment — Submitted filename: Reviews article.docx [file pone.0284520.s005.docx]

Reviews article

Points 173-179 in results page (9). (comments/ suggestion.

Mentioned the name of hospital, city.

01. During first retrospective study period there were 581 ,884 veterans reported to have had COVID-19 infection (Table1).

02. in which 74,195 (12.7%) were hospitalized and treated within the VHA hospital system.

03. the demographic characteristics of this cohort is mentioned in (Table 2).

04 the majority 179 (75.2%) had a BMI greater than or equal to 25.

05 the trend of serum PCL changes in patients of whom had multiple 183 measurements (Table 3).

06. points 268-379 discussion page (15) composers’ the chapter of discussion.

07. Explain the protein function, immunology pathway,

08 Explain the protein role in covid-19 ?.
