# Supplementary material for: Serum procalcitonin level is independently associated with mechanical ventilation and case-fatality in hospitalized COVID-19-positive US veterans–A potential marker for disease severity
Source: PLoS One. 2023 Apr 17;18(4):e0284520. doi: 10.1371/journal.pone.0284520 (PMC10109491; doi:10.1371/journal.pone.0284520)
Supplement: Attachment — Submitted filename: Response to reviewers_Final.docx [file pone.0284520.s006.docx]

**Editor-In-Chief**

Emily Chenette

Cambridge, England, UK

Dear Dr. Chenette,

Thank you for the feedback. We have reviewed the comments received and made the appropriate changes. There are given below.

1. Reviewer #1 Concern 1: While the authors addressed the first two concerns in the previous round of review, the third one, i.e., the specificity of PCT for COVID prognosis, is still not addressed.

*Response:* Combining this concern with how Reviewer 1 stated it previously, the reviewer is specifically referring to how accurately, based on a receiver operating characteristic (ROC) analysis, PCT predicts our outcomes in COVID patients. Firstly, the aim of this study was to understand the association of PCT with poor survival outcomes. We did not aim to understand the prognostic value of the PCT. Thus, we did not attempt to do the ROC analysis previously. We did convey this message in the comment. However, it appears our explanation was not satisfactory to Reviewer 1. Thus, as requested by Reviewer 1, we performed the ROC analysis, which is given below, as we felt Reviewer 1 is curious to see it. Achieving a ROC of 0.70 would require a multivariable prediction equation that includes PCT, where the equation is derived from a prospective study with PCT testing being randomized. However, the ROC area was lower (0.53-0.60).We did not feel below ROC analysis would add any further value to the manuscript and hence did not include it.

*A cursory evaluation of PCT as a prognostic indicator of ventilation and death***:** The aim of this study was not to derive a prognostic model of ventilation and death in COVID-19 patients. The results presented, however, suggest that PCT is a useful prognostic indicator of these outcomes. To support that suggestion, we used PCT by itself as the only predictor variable to obtain prognostic model test characteristics. We first limited the data to the n=10,813 patient cohort with at least two PCT laboratory values preceding ventilation, death, or discharge, whichever came first. We then used the first PCT value to predict ventilation and death. We report the area under the ROC curve when PCT is used as a continuous variable. Then, we formed a binary predictor by dichotomizing the PCT variable at the optimal cutpoint, which was the value of PCT at the point on the ROC curve closest to the upper left corner (where sensitivity=0 and 1-specificity=0). Finally, we show the test characteristics (ROC area, sensitivity, and specificity) for the binary predictor. We repeated this using the second PCT value to see if test characteristics improved, which would support the usefulness of serial measurements of PCT. The results are shown in Table R1.

Table R1. PCT test characteristics in a cohort of n=10,813 patients with at least two PCT laboratory results.

|  | PCT optimal  cutpoint | ROC  area | Sensitivity | Specificity |
| --- | --- | --- | --- | --- |
| predicting ventilation |  |  |  |  |
| first PCT | continuous | 0.56 | 0.59 | 0.54 |
| second PCT | continuous | 0.59 | 0.60 | 0.59 |
| first PCT | 0.19 | 0.56 (0.55 , 0.58) | 0.57 (0.54 , 0.59) | 0.56 (0.55 , 0.57) |
| second PCT | 0.20 | 0.59 (0.58 , 0.60) | 0.60 (0.57 , 0.62) | 0.59 (0.58 , 0.60) |
| predicting death |  |  |  |  |
| first PCT | continuous | 0.57 | 0.56 | 0.57 |
| second PCT | continuous | 0.60 | 0.61 | 0.59 |
| first PCT | 0.20 | 0.57 (0.56 , 0.58) | 0.56 (0.55 , 0.58) | 0.57 (0.56 , 0.58) |
| second PCT | 0.19 | 0.60 (0.59 , 0.61) | 0.59 (0.57 , 0.61) | 0.60 (0.59 , 0.62) |

***Discussion***: Although we provided some PCT cutpoints (< cutpoint = outcome likely not to occur ; ≥ cutpoint = outcome likely to occur) to predict ventilation or death during the hospitalization, this was only to give a sense of how predictive it is. We do not recommend these cutpoints be used in clinical practice for several reasons: the cutpoints were derived in the limited situation when an PCT test is ordered for clinical reasons and thus only apply to patients in that specific situation, the cutpoints have not been validated, the ROC area did not meet the 0.70 threshold for being a clinically useful test, and a proper prognostic model should include other variables and be derived in a prospective study.

2. Reviewer 2 concern 1: “Line 25 - include the most recent COVID-19 deaths (currently 6.8 million)”

*Response:* *Changed as suggested (current Lines 26 and 52)*

3. Reviewer 2 concern 2: “Line 52-53, appropriately cite the WHO website”

*Response:* *The* *WHO website is included as the reference [4], and all other references thereafter were renumbered.*

4. Reviewer 2 Result section:

- Line 180-181, consider including PCT levels of all groups.
- Table 1, would recommend changing the table to a flow chart.
- Table 2: Age group (unknown) I am not sure what unknown refers to as it doesn't add up to the (n) of each group. Additionally, I think it would be more informative/ clinically meaningful to report the percentages per group rather than per variable. For example, % of >/= 0.1 PCT is (38.1% in the recovered without MV vs. 21.3% in the recovered after MV... etc). Would do this to all the variables. Also, consider adding %s for all hospitalized patients in all the tables.
- Table 3: Add the number of patients in each group who had repeated PCT. Also would recommend changing the way %s are reported (similar to the above comment)

*Response*: *All of the above suggestions are now incorporated into the manuscript.*

- *Written as “This value was 3 times higher in veterans who suffered in-hospital death (0.3 (0.1-0.8) ng/ml) after receiving mechanical ventilation and 2 times higher for those who recovered after ventilation or those who perished without ventilation (0.2 (0.1-0.6) ng/ml).” Now Lines 176-178”.*
- *Table 1 is now redone into a Figure (Fig 1)*
- *Previous Table 2 is now renamed as Table 1. An explanation of “the unknown” is given in the caption.*
- *As suggested, the percentages of the group are given in previous Tables 2-3 (new Tables 3-4).*

5. Reviewer 3 concern 1: Revise Abstract, Background portion in the abstract is quite lengthy.

*Response: As suggested, the background section is now revised in the Abstract. Lines 26-30.*

6. Reviewer 3 concern 2: Concise lines 63-67

*Response: Previous lines 63-67 revised to read (now Lines 64-65), “*Amongst them, serum level markers, as well as immune cell counts, have all been associated with symptom severities, including case-fatalities [14-26].”

7. Reviewer 3 concern 3: The statement in lines 71-73 is ambiguous because not only CRP but other biomarkers are also responsible, like D-dimer.

*Response: Previous lines 71-73 revised to read, “*Previous COVID-19 studies have suggested that the blood plasma laboratory values of PCT as well as others such as CRP, D-dimer, etc.,…” – (new lines 70-71)

8. Reviewer 3 concern 4: In the 284, replace THE WORD “covid” COVID-19.

*Response: Revised as suggested.*

9. Reviewer 3 concern 5: Most of the statements in the discussion should briefly be discussed; it is quite lengthy for readers:

*Response: Revised as suggested. The race analysis section did not add any value to the manuscript. Hence, we removed race references from all sections to make the manuscript more concise and relevant.*

Kind Regards,

Sujee Jeyapalina, PhD

Division of Plastic Surgery, Department of Surgery

University of Utah School of Medicine

Salt Lake City, UT 84132

Email: Sujee.jeyapalina@hsc.utah.edu

Tel: 801-582-1565 ext. 1854

Fax: 801-588-5939
